# Development of DNA-Based Lateral Flow Assay for Detection of LDLR Gene Mutation for Familial Hypercholesterolemia

**DOI:** 10.21315/mjms2024.31.3.6

**Published:** 2024-06-27

**Authors:** Lina Khalida Saidi, Zam Zureena Md Rani, Siti Aishah Sulaiman, Rahman Jamal, Aziah Ismail, Anis Amirah Alim, Sharipah Nadzirah Syed Ahmad Ayob, Chang Fu Dee, Azrul Azlan Hamzah, Nor Azian Abdul Murad

**Affiliations:** 1 UKM Medical Molecular Biology Institute (UMBI), Universiti Kebangsaan Malaysia, Kuala Lumpur, Malaysia; 2 Institute for Research in Molecular Medicine (INFORMM), Universiti Sains Malaysia, Kelantan, Malaysia; 3 Institute of Microengineering and Nanoelectronics (IMEN), Universiti Kebangsaan Malaysia, Selangor, Malaysia; 4 Institute of Nano Electronic Engineering (INEE), Universiti Malaysia Perlis, Perlis, Malaysia

**Keywords:** lateral flow assay, familial hypercholesterolemia, DNA mutations, single nucleotide polymorphism, genetic screenings

## Abstract

**Background:**

The techniques for detecting single nucleotide polymorphisms (SNP) require lengthy and complex experimental procedures and expensive instruments that may only be available in some laboratories. Thus, a deoxyribonucleic acid (DNA)-based lateral flow assay (LFA) was developed as a point-of-care test (POCT) diagnostic tool for genotyping. In this study, single nucleotide variation (E101K) in the low-density lipoprotein receptor *(LDLR)* gene leading to familial hypercholesterolemia (FH) was chosen as a model.

**Methods:**

Hypercholesterolemic individuals (*n* = 103) were selected from the Malaysian Cohort project (UKM Medical Molecular Biology Institute) while the control samples were selected from the Biobank (UKM Medical Molecular Biology Institute). The DNA samples were isolated from whole blood. Polymerase chain reaction (PCR) amplification process was performed using bifunctional labelled primers specifically designed to correspond to the variant that differentiates wild-type and mutant DNA for visual detection on LFA. The variant was confirmed using Sanger sequencing, and the sensitivity and specificity of the LFA detection method were validated using the Agena MassARRAY^®^ technique.

**Results:**

Out of 103 hypercholesterolemic individuals, 5 individuals (4.8%) tested positive for E101K, *LDLR* mutation and the rest, including healthy control individuals, tested negative. This result was concordant with Sanger sequencing and Agena MassARRAY^®^. These five individuals could be classified as Definite FH, as the DNA diagnosis was confirmed. The sensitivity and specificity of the variant detection by LFA is 100% compared to results using the genotyping method using Agena MassARRAY^®^.

**Conclusion:**

The developed LFA can potentially be used in the POC setting for detecting the E101K variant in the *LDLR* gene. This LFA can also be used to screen family members with E101K variant in the *LDLR* gene and is applicable for other SNP’s detection.

## Introduction

Genotyping refers to techniques directly assessing known or novel mutations or single nucleotide polymorphisms (SNPs) (1). The increased prevalence of diseases due to molecular defects or mutation in the gene that causes the disease has created a significant demand for developing a quick and simple diagnostic test for nucleic acid-based genotyping applications. Several genotyping techniques have been developed for the detection of SNPs involving quantitative polymerase chain reaction (PCR) analysis (2), MassARRAY^®^ (3), custom-made microarrays (4), amplification-refractory mutation system (5), denaturing high-performance liquid chromatography (6), single-strand conformation polymorphisms (7), high-resolution melting analysis (8) and real-time PCR (9). However, these techniques require lengthy and complicated experimental procedures and expensive instruments that may not be available in many laboratories. Therefore, a simple and rapid genotyping technique is essential. The above-mentioned limitations can be overcome by providing a rapid point-of-care test (POCT) diagnostic tool specific for nucleic acid detection. POCT is a term used to describe laboratory tests that can be performed by non-laboratory personnel and can be performed at locations outside the laboratory (10). The development of POCT for nucleic acid detection still requires three main steps: i) sample preparation, ii) amplification and iii) detection process. However, the size, cost and complexity can be improved to support POCT criteria (11). One of the potential diagnostic tools to be used as POCT equipment is lateral flow assay (LFA).

LFA is a device platform composed of components including a sample pad, conjugate pad, nitrocellulose membrane and adsorbent pad assembled in a backing pad (12). LFA can detect and quantify target analytes, where test results can be displayed within 5 min–30 min after the sample is placed on the assay device. The development cost is USD0.10–USD3.00 for a single test (13). The development of LFA began with the latex agglutination test performed by Plotz and Singer in 1956, but its application was only first commercialised in the 1980s for pregnancy diagnosis (14). Now, pregnancy test LFA is widely used for home pregnancy tests worldwide. Due to the low development cost and faster turnaround time, many studies have used the nucleic acid LFA method as a diagnostic tool to diagnose disease and genotype drug-resistance genes involved in infectious diseases (15–20).

The LFA design consists of several important components mounted on a plastic backing pad, which are the sample pad, conjugate pad, nitrocellulose membrane and absorption pad (12). The backing pad on the LFA provides mechanical support to the LFA device. Sample pads and conjugate pads are usually made of fibre glass. The sample pad is where the sample is filtered and the target analyte present in the sample will move through capillary action through the conjugate pad, which has been immobilised with a specific antibody bound with coloured or fluorescence label. Then, the target analyte is bound to the conjugated antibody, and the sample moves further to the nitrocellulose membrane, where the test and control zones are located. The test zone was immobilised with a specific antibody to capture the target analyte bound to the conjugated antibody. The presence of conjugated antibody-target analyte-antibody on the test zone causes a red line to appear and it can be seen with the naked eye or measured using a detector. The control zone has also been immobilised with a non-specific antibody to capture the conjugated antibody and the appearance of a red line on this zone indicates that the test is working correctly and prevents false positive results. The excess sample will then be absorbed by an absorption pad, usually composed of high-density cellulose, located at the end of the LFA strip.

In this study, a DNA-based LFA was developed and a monogenic autosomal dominant disease, familial hypercholesterolemia (FH), with the E101K variant in the low-density lipoprotein receptor (*LDLR*) gene, was chosen to be the model for LFA detection. This variant causes single nucleotide change (G > A) and this may cause the respective functional areas of the *LDLR* gene to be deleted or functionally impaired (21), thus affecting the LDL cholesterol (LDLC) metabolism and consequently causing elevated LDLC levels in the blood (22). Untreated FH patients will increase the risk of developing premature coronary heart disease.

## Methods

### Sample Size Calculation, Sample Collection and DNA Isolation

The sample size was calculated based on the formula of specificity (sP) and sensitivity (sN) for the diagnostic test (with consideration of FH prevalence) (23). The sP and sN of the LFA were presumed to be 95%, and the desired width of the 95% CI was 0.05. The prevalence of FH in the Malaysian population was 8.3% (24). Based on these assumptions, this study’s minimum number of samples to achieve 95% sP and sN was 879 and 80, respectively. However, because this study is a pilot study, only 10% of the total sample size will be used in this study. Nevertheless, the number of samples was increased to 103 samples for cases and 60 samples for controls. For cases, participants with a total cholesterol level of > 5.2 mmol/L and LDL cholesterol level of > 3.4 mmol/L (based on laboratory reference range) (25) from The Malaysia Cohort (TMC) project were selected. The healthy control cohort was selected from healthy individuals with a total cholesterol level of < 5.2 mmol/L from the lipid profile test (based on the laboratory reference range) (25). The DNA extraction from the blood sample was performed by the TMC using the NucleoMag^®^ DNA kit (Macherey-Nagel, Germany) on a semi-auto DNA extraction machine, King Fisher Duo Prime Purification (ThermoFisher Scientific, USA) based on the protocol distributed by the manufacturer.

### GBlock Gene Fragments and Primer Design

Gene fragments containing E101K variant in the *LDLR* gene or called gBlocks gene fragments was synthetically designed. The gBlock gene fragments consist of wild-type and mutant fragments specific for the E101K variant. The whole sequence of the *LDLR* gene specific to the E101K variant was obtained from the NCBI GenBank at the following website: (https://www.ncbi.nlm.nih.gov/genbank/). The gBlock gene fragments were purchased from Integrated DNA Technologies, USA. The gBlock gene fragments were resuspended according to the protocol distributed by the manufacturer. All primers (Table 1) used in this study were designed using NCBI Primer-Blast (https://www.ncbi.nlm.nih.gov/tools/primer-blast/). All primers were attached to specific tags at the 5′ terminal: i) digoxigenin (DIG) (wild-type forward primer); ii) carboxyfluorescein (FAM) (mutant forward primer); and iii) Biotin (reverse primer). All tagged primers were purchased from Azenta Inc., China.

### Preparation of LFA Strip

**Figure 1:**
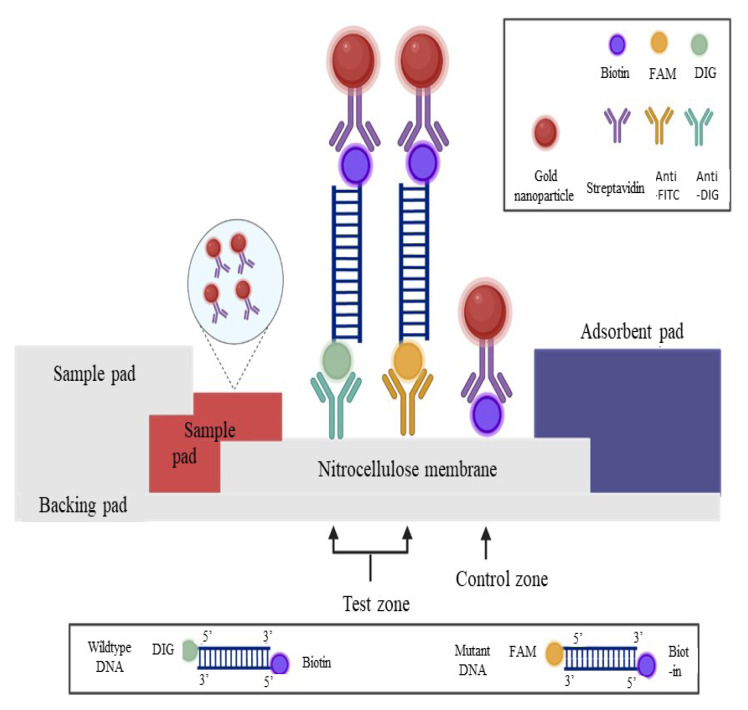
Schematic illustration of the LFA to visualise PCR amplicons using immobilised capture reagents on the nitrocellulose membrane. Wild-type DNA amplicon is labelled with DIG and biotin and is captured by anti-DIG antibody on the test zone; Mutant DNA amplicon is labelled with carboxyfluorescein (FAM) and biotin and is captured by anti-FITC antibody on test zone. The accumulation of streptavidin-colloidal gold conjugate in the respective zones produced visible red lines. The excess streptavidin-colloidal gold conjugate is captured by biotin on the control zone to validate the LFA. The figure was created using Biorender.com online software

The sample pad (No. 319) (Ahlstrom, Finland), conjugate pad (No. 8964) (Ahlstrom, Finland), nitrocellulose membrane (CN95) (Sartorius, Germany) and absorbent pad (C048) (Millipore, USA) were assembled on a plastic adhesive backing card assay strip supporter (G&L Precision Die-cutting, Netherlands) to fabricate the LFA. The strip was cut into 5 mm × 63 mm sizes using an automatic strip cutter (Kinematic Automation, USA). The conjugate pad was immobilised with streptavidin antibody-conjugated gold nanoparticles (40 nm) (Kestrel BioSciences, USA) in the presence of trehalose as a stabiliser. Three capture reagents were immobilised on a nitrocellulose membrane to produce a test zone and a control zone: i) mouse monoclonal anti-fluorescein isothiocyanate (FITC) (Sigma Aldrich, USA) as the target to normal DNA in the test zone I; ii) anti-DIG (Roche, Germany) as the target to mutant DNA in test zone II; iii) and goat anti-mouse IgG (whole molecule)-biotin antibody (Sigma Aldrich, USA) in control zone. The schematic diagram of LFA is shown in Figure 1.

### PCR Assay and Visual Detection of PCR Amplicon Using LFA Strip

A PCR assay was performed on all gBlock gene fragments, hypercholesterolemic individuals and healthy control samples. The PCR assays were performed by preparing the reaction mixture containing HotStarTaq Master mix (Qiagen, Germany), 10 μM of forward (wild-type and mutant) and reverse primer (Integrated DNA Technologies, USA), RNase-free water (Qiagen, Germany) to make the final volume of 16 μL. PCR was performed using 5 μL of gBlock/DNA sample in a thermal cycler (Bio-Rad, USA) with the following cycling parameters of 95 °C for 15 min, followed by 35 cycles of denaturation at 95 °C for 30 s, annealing at 67 °C for 30 s and elongation at 72 °C for 30 s, with a final extension at 72 °C for 10 min. The PCR product was visualised by UV through a 1.2% (w/v) agarose gel in 1× Tris-Borate-EDTA (TBE) buffer stained with ethidium bromide (10 μg/mL), electrophoresed at 80 V for 60 min. A 100 bp DNA molecular weight ladder was used in the gel as a base pair size reference.

**Figure 2:**
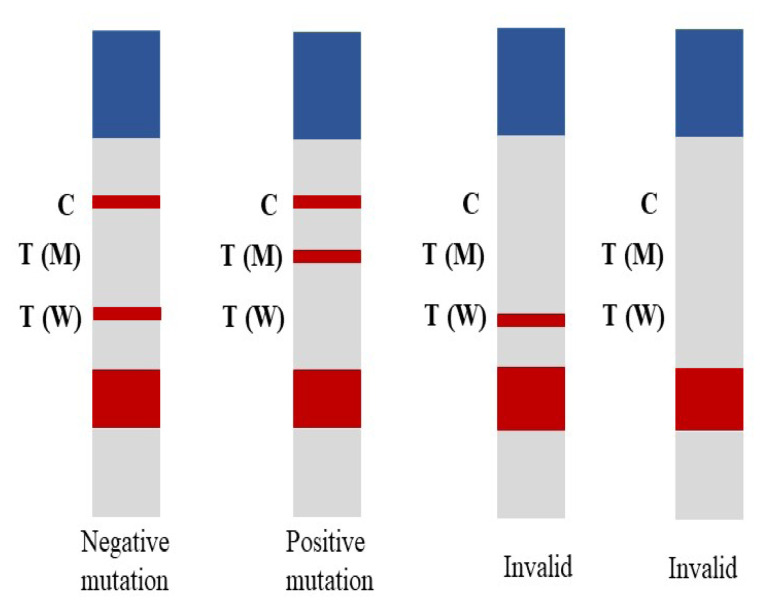
Interpretation of LFA result

For LFA, 10 μL of the PCR product was pipetted and placed on the sample pad of the LFA strip. Then, two drops of strip buffer were added to the sample pad and left for 5 min. The running buffer contained 1% bovine serum albumin (BSA) and 0.05% Tween-20 in phosphate-buffered saline, pH 7.4. Then, one drop of strip buffer was added to the sample pad and left for 5 s. This step was repeated three times. The mixture moves from the sample pad to the absorption pad by capillary action. After 15 min, the results were read based on the presence of a red line on the LFA strip. The result interpretation is shown in Figure 2.

### Mutation Validation Using Sanger Sequencing

GBlock gene fragments and representative samples from hypercholesterolemic individuals and healthy control samples were subjected to Sanger Sequencing to confirm the E101K variant. The product obtained from PCR amplification was first purified using the QIAquick PCR Purification kit (Qiagen, Germany) according to the manufacturer’s protocol. Subsequently, the reaction mixture was prepared for cycle sequencing using BigDye V3.1 terminator (Applied Biosystem, USA), BigDye sequencing buffer (Applied Biosystem, USA), exon 3 forward primer (Integrated DNA Technologies, USA) and RNAse free water (Qiagen, Germany) to make a final volume of 9.0 μL. The cycle sequencing was performed using 1.0 μL of pure PCR products in a 96-well plate in a thermal cycler using the following cycling parameters: 96 °C for 1 min, followed by 25 cycles of denaturation at 96 °C for 10 s, annealing at 50 °C for 5 s and elongation at 60 °C for 2 min. Next, the purification process was performed on the cycle sequencing product using the ethanol precipitation method. DNA sequencing was performed using the 3500 Genetic Analyzer (Applied Biosystem, USA). The DNA sequence was analysed using the Sequence Scanner version 2.0 software (Applied Biosystem, USA) and confirmed with the NCBI-blast database (https://blast.ncbi.nlm.nih.gov/Blast.cgi). The wild-type and mutant nucleotide peak was visualised using Chromas version 2.6.6 (Technelysium Pty. Ltd., Australia).

### Technical Validation of LFA Using Agena MassARRAY^®^ Method for Specificity and Sensitivity

All gBlock gene fragments, hypercholesterolemic individuals and healthy control samples used for the LFA detection method were subjected to Agena MassARRAY^®^ for technical validation. Briefly, a panel assay was designed involving the primer design for the PCR process and the iPLEX reaction for the variant of interest (E101K) using the MassARRAY^®^ Assay Design Suite (ADS) software (Agena Bioscience, USA). Then, the PCR amplification, shrimp alkaline phosphatase (SAP) treatment and primer extension reaction were performed according to the manufacturer’s instructions using iPLEX Pro assay (Agena Bioscience, USA). Briefly, the PCR reaction was carried out by preparing the reaction mixture containing 10× PCR buffer with 20 mM MgCl_2_, 25 mM dNTP, 25 mM MgCl_2_, 0.5 uM Primer Mix and PCR enzyme (PCR Accessory and Enzyme Kit; Agena Bioscience, USA) to make the final volume of 5 μL. The PCR amplification was performed using 2 μL of DNA sample with the following cycling parameters of 95 °C for 2 min, followed by 45 cycles of denaturation at 95 °C for 30 s, annealing at 56 °C for 30 s and elongation at 72 °C for 60 s, with a final extension at 72 °C for 5 min. Unincorporated dNTPs were deactivated using SAP enzyme and buffer. Then, the PCR product was subjected to primer extension reaction consisting of iPLEX Pro buffer, iPLEX Pro Enzyme, termination mix and extended primer (iPLEX Pro Kit; Agena Bioscience, USA) and then underwent the following cycling parameters of 94 °C for 30 s, followed by 40 cycles of 95 °C for 5 s, 5 inner cycles of 52 °C for 5 s, 80 °C for 5 s, and finally 72 °C for 3 min (iPLEX pro kit; Agena Bioscience, USA). After desalting, the product was transferred from the 96-well microtiter plate to the SpectroCHIP^®^ CPM 96 array (Agena Bioscience, USA) using MassARRAY^®^ system with Chip Prep Module and analysed using MassARRAY^®^ analyser (Agena Bioscience, USA). MALDI-TOF MS captured mass signals for the variant and the data obtained from the assay was analysed using TYPER 4.0 software (Agena Bioscience, USA).

Next, the sP and sN of the LFA method as an index test were calculated according to the sP and sN formula for diagnostic tests (26), as in Table 2.

### Statistical Analysis

Statistical analysis was performed to analyse the data. Categorical variables were defined as values and percentages and compared using the chi-square test (χ^2^). Continuous variables were defined as mean ± standard deviation (SD) and compared using the Mann-Whitney U test. The data was collected and analysed using SPSS version 22.0 (SPSS Inc., Chicago IL, USA). A *P*-value less than 0.05 was considered significant, indicating a significant difference or association between the compared groups.

## Results

### Demographic Data of Participants Involved in this Study

The demographic data involves age, gender and race, and clinical findings, including medical history and biochemical data involving lipid profile parameters, are shown in Table 3. As for hypercholesterolemic individuals, for medical history, 49.5 % of them have a family history of cardiovascular disease (CVD).

### PCR Assay, LFA Visual Detection and Mutation Conformation Using Sanger Sequencing for E101K Variant on gBlock Gene Fragment Samples

**Figure 3:**
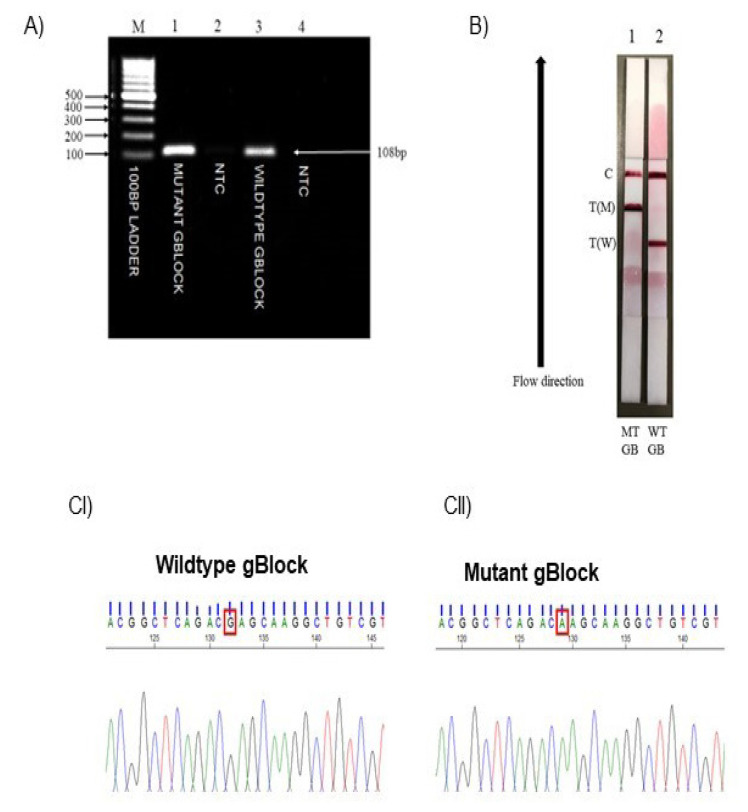
**(A)** Agarose gel electrophoresis analysis of PCR amplicon for E101K variant, *LDLR* gene for gBlock gene fragment samples. Samples were analysed through a 1.2% (w/v) agarose gel in 1×TBE stained with ethidium bromide. The gel was run at 80 V for 60 min. Bands were visualised by UV transillumination. The 108 bp band represents the amplified product. Lane M: DNA marker; Lane 1: mutant gBlock gene fragment; Lane 2: non-template control (mutant); Lane 3: wild-type gBlock gene fragment; Lane 4: non-template control (wild-type). **(B)** Variant detection using LFA for mutant and normal gBlock gene fragments for E101K variant. Strip 1 (mutant gBlock gene fragment sample): positive; Strip 2 (wild-type gBlock gene fragment sample): negative. Black arrows indicate the direction of the flow. **(CI)** Sequencing analysis of exon 3, E101K variant, *LDLR* gene. Wild-type gBlock gene fragment sample; **(CII)** mutant gBlock gene fragment sample. The red box on the sequence analysis showed a nucleotide base changes from G to A, which causes an amino acid change from glutamic acid (E) to lysine (K) at codon 301 and protein 101 or the E101K variant. The presence of one peak (green/black) on the sequence analysis indicates a homozygous form

Wild-type and mutant gBlock gene fragment samples were used to test the functionality of the LFA (Figure 3). The developed LFA consists of two test lines representing wild-type (W) and mutant (M) and one control (C) line. When samples were applied on the sample pad, the samples moved up towards the absorption pad through capillary action. Results can be observed within 15 min after sample exposures by red lines on the test and control zone, respectively. The mutant gBlock gene fragment sample (strip 1) tested positive for the E101K variant due to the presence of two lines on the M and C lines.

On the other hand, the wild-type gBlock gene fragment sample (strip 2) tested negative for the variant on the W and C lines. As for the DNA analysis, it was performed using Sequence Scanner software version 2.0. The sequence and peak for wild-type and mutant gBlock samples were shown below (Figure 3 CI and CII), and it focused on the single nucleotide change that represents the E101K variant located at the exon 3 of the *LDLR* gene. The red box on the sequence analysis represents the location of the single nucleotide change where the nucleotide base changes from G to A, representing the E101K variant.

### LFA Visual Detection and Mutation Confirmation Using Sanger Sequencing for E101K Variant on Hypercholesterolemic Individuals and Healthy Control Samples

**Figure 4:**
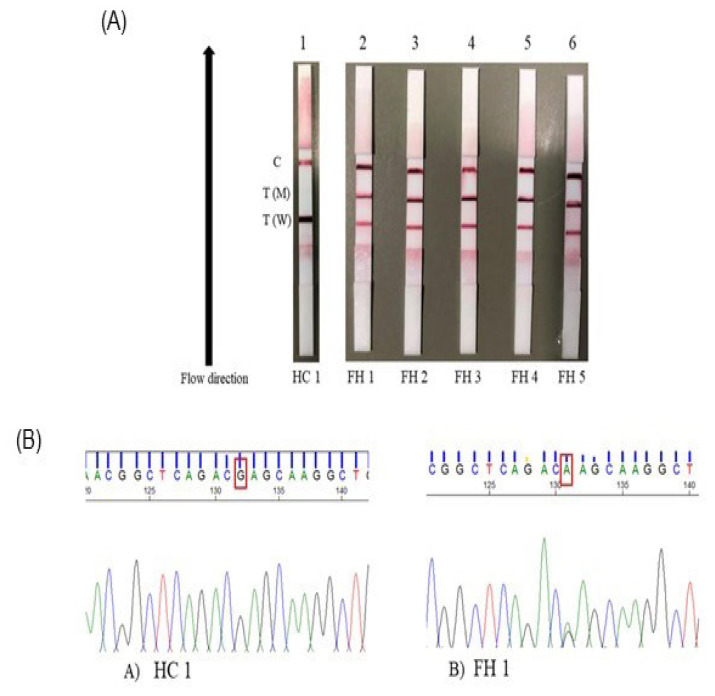
**A)** Mutation detection of E101K, *LDLR* gene using LFA. Strip 1 (Healthy control, HC,1): negative; Strip 2 (FH 1), strip 3 (FH 2), strip 4 (FH 3), strip 4 (FH 4), strip 6 (FH 5): positive. Black-coloured arrows indicate the flow direction of the samples. **(B)** Sequencing analysis of exon 3, E101K variant, *LDLR*. A) HC 1, B) FH 1. The red box showed a nucleotide base change from G to A. This causes an amino acid change from glutamic acid (E) to lysine (K) at codon 301 and protein 101 or the E101K variant. The presence of two peaks (green and black) on the sequence analysis indicates heterozygous forms

The result for LFA detection on representative hypercholesterolemic individuals and healthy control samples is shown in Figure 4(A). Among 103 hypercholesterolemic individuals, 5 (4.8%) samples tested positive for the E101K variant and the rest tested negative. All 60 healthy control samples tested negative for the variant. Test strip 1 (healthy control 1) was negative for variant due to the presence of two lines on the W and C lines, while test strips 2 (FH1), 3 (FH 2), 4 (FH 3), 5 (FH 4) and 6 (FH 5) were positive for variant due to the presence of three lines on the W, M and C lines. After LFA detection, the representative samples were subjected to Sanger Sequencing to confirm the E101K variant. The DNA analysis was performed using Sequence Scanner software version 2.0; the representative result is shown in Figure 4(B) below. The sequence and peak analysis focused on the single nucleotide change that represents the E101K variant located at the exon 3 of the *LDLR* gene. The location of the single nucleotide change was indicated by the red box on the sequence analysis where the nucleotide base changes from G to A, representing the E101K variant.

### Technical Validation of LFA Using Agena MassARRAY^®^ Method and Specificity and Sensitivity Calculation

**Figure 5:**
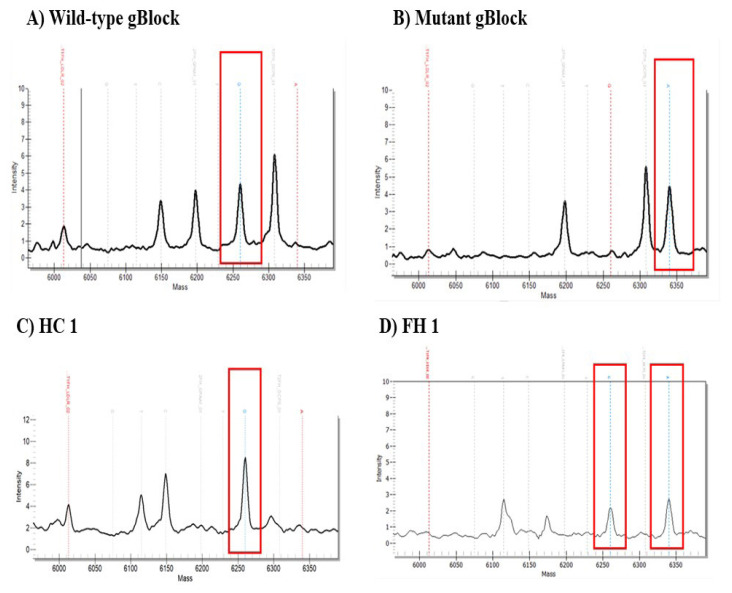
MassARRAY^®^ spectrum analysis for gBlock gene fragment and representative clinical samples for E101K, *LDLR* gene. **A)** Wild-type gBlock: negative. **B)** Mutant gBlock: positive. **C)** Healthy control, HC 1: negative. **D)** FH 1: positive. The presence of one peak (red boxes) on the spectrum analysis indicates homozygous form, while the presence of two peaks (red boxes) on the spectrum analysis indicates heterozygous form

The MassARRAY^®^ results of gBlock gene fragments and representative samples are shown in Figure 5 below. Based on the result, wild-type gBlock was negative and mutant gBlock was positive for the E101K variant due to one peak representing G and A nucleotide, respectively (red box). As for representative samples, the HC 1 sample was negative for the E101K variant due to one peak representing G nucleotide (red box). On the other hand, FH 1 was positive for the E101K variant due to two peaks representing G and A nucleotide bases (red boxes). No false negative and false positive result was detected from the overall Agena MassARRAY^®^ result compared to the LFA detection method. Thus, the LFA detection method has an overall sP and sN of 100% compared to Agena MassARRAY^®^ as the reference test.

## Discussion

In this study, the E101K (G > A) variant in the *LDLR* gene, which causes Familial hypercholesterolemia (FH), was chosen as the target. This variant has been reported in the Malaysian population with 7.1% (27). This variant was also predicted to be pathogenic based on in silico analysis that results in amino acid changes, or missense type of variant in which the amino acid changes from medium-sized, acidic amino acid (glutamic acid) to large-sized, basic amino acid (lysine). This variant is located at the second disulphide-rich repeat in the receptor protein binding domain and impacts the processing and intracellular transport of the generated protein (27). Moreover, some studies have suggested a damaging outcome of this variant (28, 29).

To ensure that the primers used are specific to the single nucleotide change that represents the E101K variant, the forward primer was designed with the 3′ terminal of the primer corresponding to the E101K variant in the *LDLR* gene. The mutant forward primer was designed to end with an A at the 3′ terminal of the primer, and the wild-type forward primer ended with a G at the 3′ terminal of the primer. The reverse primer used is similar for both wild-type and mutant DNA. These primers were directly tagged with specific tags to enhance the targeted amplicon’s specificity during LFA detection.

Principally, the PCR product with a couple of drops of running buffer was applied on the sample pad of the LFA. Then, the mixture flowed towards the adjacent component, the conjugate pad. Next, it released the dried streptavidin-colloidal gold nanoparticles conjugate that was immobilised on the conjugate pad and consequently bound to the 5′-biotin labelled of the PCR amplicons. This study has used colloidal gold nanoparticles as the label since it has been primarily used in other studies due to their excellent optical properties, high affinity binding and ease of being functionalised towards biomolecules (12, 30–32). After that, the mixtures flowed up towards the membrane of the strip by capillary action where the PCR amplicons that have been labelled with 5′ FAM (mutant DNA) and 5′ DIG (wild-type DNA) bound to capture antibodies involving anti-FITC and anti-DIG that have been immobilised on the test zones to form a complex. The naked eye can visualize these as red lines due to the accumulation of streptavidin-colloidal gold nanoparticle conjugate. The excess streptavidin-colloidal gold nanoparticles attached to biotin immobilised on the control zones, producing red lines, indicating that LFA is functioning correctly.

Based on the LFA result (Figures 3B and 4A), the result was different between mutant gBlock gene fragments and hypercholesterolemic individuals’ samples that tested positive for the E101K variant. One line appeared at the test zones for the mutant gBlock gene fragment sample, while two lines appeared at the test zones for FH individual samples. It is because the gBlock gene fragments DNA sequence was synthetically designed to contain the E101K homozygous variant in the *LDLR* gene. The two lines on the test zones for positively tested hypercholesterolemic individuals indicate that the E101K variant is in heterozygous form. These LFA results were validated by Sanger sequencing by the presence of two peaks at the location of the variant representing heterozygous form for representative hypercholesterolemic individuals. Sanger sequencing was performed in this study to confirm the E101K variant detected by LFA. This method is commonly used in the diagnostic lab and is a gold standard for confirming variant identified in other methods (33, 34). Out of 103 hypercholesterolemic individuals, 5 (4.8%) tested positive using the LFA method (Figure 4), which concords with the result obtained from Sanger sequencing and Agena MassARRAY^®^. These individuals could be classified as Definite FH, as the DNA diagnosis has been confirmed. The LFA could be used for cascade screening to identify FH in the family members for early diagnosis. In addition, statin treatment can be started to reduce the risk of cardiovascular diseases.

The sP and sN of the LFA as an index test were calculated, and the result showed that LFA has 100% sP and sN compared to the Agena MassARRAY^®^ method. Agena MassARRAY^®^ was used to validate the result obtained from LFA. This method allows the genotyping of a custom set of SNPs at specific locations in the genome with high sP and sN while remaining cost-effective (35). This shows that the LFA method is sensitive and specific, and it can potentially be used as a diagnostic tool for SNP genotyping, which is associated with disease and has an impact on pharmacogenomics. However, LFA is more useful in diagnosing genetic diseases with homogeneous variants, such as thalassemia disease, and for infectious diseases such as COVID-19 (36), although a systematic review analysis based on preliminary data and pre-print papers has reported that the performance of the LFA has dropped with regard to the newer SARS-CoV-2 variants, the omicron variant, when compared to previous delta variant due to manufacturer dependent and lower viral loads in asymptomatic individuals (37). For diseases with heterogeneous variants, several techniques, such as whole exome sequencing and targeted sequencing, could be used to identify the variant and subsequent analysis using LFA for the family member can be done (34, 38, 39).

This study has compared the time efficiency and cost-effectiveness of the method involving LFA, Sanger sequencing and Agena MassARRAY^®^. Regarding cost-effectiveness, the cost per reaction for developing the DNA-based LFA test for the E101K variant in the *LDLR* gene is lower than Sanger sequencing, which is RM30.00 and RM31.50, respectively. As for the Agena MassARRAY^®^ method, the cost for one iPLEX Pro kit is RM23,000.00 with the capability of analysing 960 samples. Therefore, the estimated cost per reaction is RM23.96 for a 96-well plate. As for time efficiency, the LFA test is twice as fast as Sanger sequencing, requiring only 210 min compared to 435 min. This is because the LFA test does not involve DNA denaturation and lengthy analysis time like most existing sequencing methods. Furthermore, the LFA method does not require trained personnel to operate complex instruments, as it does not involve complex genotyping instruments. In the future, gel electrophoresis will not be required after the PCR process, and the PCR amplification products can be directly analysed for variant detection using the LFA test.

Furthermore, in terms of the cost of producing one LFA strip unit, the required cost is RM26.00. The cost of producing LFA strips is expected to decrease in the future, as seen with the COVID-19 pandemic, where the cost of producing COVID-19 LFA strips decreased from > RM40.00 to < RM10.00. However, although the LFA method is seen to assist in detecting the E101K variant in the *LDLR* gene with high sP and sN, DNA sequencing remains the standard method for identifying variants associated with FH, especially in identifying index patients. The use of LFA test can be utilised for variant screening in family members who are potentially affected by FH after the variant in the index case is identified using genetic testing because it is easier to use, cost-effective and provides rapid analysis time.

Many other genotyping methods have been developed for SNPs detection. However, the methods mostly require highly sophisticated, expensive instrumentation and tedious procedures (40). For example, SNP genotyping using Taqman assay requires a high cost of probes and reagents, which can accelerate the cost of genotyping (41). LFA has continued to gain attention in the research world since its first development, particularly for rapid testing due to shorter turnaround times. For example, a study has been conducted on developing a reverse transcriptase isothermal amplification nucleic acid LFA (RT-RPA-NALF) as a rapid test for detecting Porcine epidemic diarrhoea virus using specific primers and probes. The reverse transcriptase polymerase chain reaction method was used to validate the results. The study results showed that the RT-RPA-NALF can detect the amplicon virus within 25 min at a temperature of 42 °C, with a sP and sN of 100% and 95.65%, respectively, with a 98.33% coincidence rate (42).

For future improvements, the work can focus on improving the amplification method to improve the efficiency and effectiveness of the developed LFA assay. Other than that, the LFA can also be designed for other disease models, particularly in the identification of variant for diagnosis of diseases and SNPs with pharmacogenetics impact.

## Conclusion

This study demonstrated that LFA could accurately detect the presence of the E101K variant in the *LDLR* gene and potentially be used as a diagnostic test for SNP detection with 100% sP and sN. In total, 4.8% of hypercholesterolemic individuals could be classified as definite familial hypercholesterolemia (FH).

## Figures and Tables

**Table 1:** Primers used in this study

Sequences (5′-3′)	5′-Label	Size (bp)
Forward primer (wild-type): DIG-TGCGACAACGGCTCAGACG	DIG	
Reverse primer (mutant): FAM-TGCGACAACGGCTCAGACA	FAM	108
Reverse primer: Biotin-AGGACCCCGTAGAGACAAAGT	Biotin	

**Table 2:** sP and sN calculation

	Agena MassARRAY^®^	

	Tested positive	Tested negative
LFA test		
Tested positive	TP	FP
Tested negative	FN	TN
	Sensitivity (sN):	Specificity (sP):
	TP/(TP+FN)	TN/(TN+FP)

Notes: TP = true positive; FP = false positive; FN = false negative; TN = true negative

**Table 3:** Demographic, clinical findings and biochemical data for hypercholesterolemic individuals and healthy control samples

Characteristics	Hypercholesterolemic individuals (*n* = 103)	Healthy control (*n* = 60)	*P*-value
Demographic			
Age (years old)*^b^	53.9 (6.1)	40.4 (17.6)	< 0.001
Gender *n* (%)^a^			< 0.18
Male	43 (41.7)	18 (30.0)	
Female	60 (58.3)	42 (70.0)	
Race			< 0.001
Malay	103 (100.0)	42 (70.0)	
Non-Malay	–	18 (30.0)	
Lipid profile^b^			
TC (mmol/L)*	10.0 (0.9)	4.5 (0.3)	< 0.001
LDLC (mmol/L)*	7.8 (0.9)	2.6 (0.5)	< 0.001
HDLC (mmol/L)*	1.3 (0.3)	1.3 (0.3)	< 0.86
TG (mmol/L)*	1.9 (0.7)	1.1 (0.6)	< 0.001
Medical history			
Family history of hyperlipidemia *n* (%)	7 (6.8)	–	
Family history of CVD *n* (%)	51 (49.5)	–	
Individual history of hyperlipidemia *n* (%)	24 (23.3)	–	
Individual history of CVD *n* (%)	5 (4.9)	–	

Notes:

* Data stated as mean (SD);

a Chi-square test was used;

b Mann-Whitney U test was used
